# Awareness and Reporting of Antiretroviral Adverse Events Among Clients and Health-Care Providers at a Referral Hospital in Moshi, Northern Tanzania: A Cross-Sectional Study

**DOI:** 10.24248/EAHRJ-D-18-00023

**Published:** 2019-11-29

**Authors:** Mohamed Kiwanuka, Florida J Muro, Pius J Alloyce, Eva P Muro

**Affiliations:** a Kilimanjaro Christian Medical University College, Moshi, Tanzania; b Department of Epidemiology & Biostatistics, Institute of Public Health, Kilimanjaro Christian Medical University College, Moshi, Tanzania; c Reproductive Health Clinic, Kilimanjaro Christian Medical Centre, Moshi, Tanzania; d Department of Pharmacology, Kilimanjaro Christian Medical University College, Kilimanjaro, Tanzania; e Department of Pharmacy, Kilimanjaro Christian Medical Centre, Moshi, Tanzania

## Abstract

**Background:**

Pharmacovigilance is a means of ensuring drug safety, and thus it ensures that the risks associated with medication adminstration and consumption do not outweigh the benefits. Antiretroviral therapy (ART) for HIV care and treatment has reduced mortality and morbidity, but adverse drug reactions (ADRs), which can lead to treatment failure, remain a concern. In 2015 in Tanzania, 688,800 adults were taking ART. All health-care providers are required to report all suspected ADRs seen or reported by their patients using yellow forms available at all care and treatment centres in Tanzania. However, the actual practice of reporting is not taking place. This study aimed to explore the patients’ knowledge and HIV/AIDS health-care providers’ reporting of ART adverse events at Kilimanjaro Christian Medical Centre (KCMC).

**Methods:**

A cross-sectional study using a semi-structured questionnaire was conducted between June and July 2016 within HIV, dermatology, and infectious disease clinics at KCMC. All health-care providers providing HIV services within these clinics completed a questionnaire. Means and standard deviations were used to summarise the numerical data with normal distributions (age of patients), while numerical data that were not normally distributed (duration on ART) were summarised using medians and ranges. Frequencies and percentages were used to summarise categorical variables.

**Results:**

All 63 health-care providers agreed that ADR reporting was necessary. Forty-six (73%) were aware of the national ADR reporting system, but only 32 (50.8%) reported having received training on pharmacovigilance. Only 4 (6.3%) of all health-care providers reported always filling the ADR report forms; 27 (42.9%) rarely filled the forms, and 32 (50.8%) reported having never filled an ADR reporting form. Training on pharmacovigilance had a positive influence on ADR reporting. Lack of motivation, uncertainty about reporting procedures, lack of time, unavailability of reporting forms, and ignorance were the major factors affecting reporting among health-care providers.

**Conclusion:**

The majority of health-care providers were aware of the need and importance of ADR reporting and the national pharmacovigilance system. However, ART adverse events are underreported. More effort is needed to strengthen the continuous reporting of ADRs by providing continuous education to health-care providers; this will lead to their active participation in pharmacovigilance.

## INTRODUCTION

Antiretroviral therapy (ART) for the treatment of HIV infection has improved steadily after the introduction of combination therapy.^1^ In 2016, more than half (53%) of people living with HIV (PLHIV) had access to life saving treatment. The increase in the number of PLHIV accessing ART globally has occurred rapidly: from 7.5million in 2010 to 17 million in 2015before reaching 19.5 million in 2016.^2^ However, this significant improvement in ART use has been accompanied by an increase in the number of adverse drug reactions (ADR). ADRs significantly affects quality of life, and have been associated with acute hospital admissions ^3–5^ and prolonged length of hospital stay, increased economic burden and increased death. Awareness of ADR reporting procedures among patients and health-care providers is key to ensuring safety of prescribed drugs.

In 2016, there were 1.4 million PLHIV in Tanzania with an estimated prevalence of 4.7%.^6^ The HIV prevalence among adults (⁥15 years of age) in Tanzania varies geographically ranging from 11.4 % in Njombe to <1% in Lindi and Zanzibar.^7^ The current HIV prevalence among adults (15–49 years) in Kilimanjaro region is estimated to be 2.6%. In 2016, in Tanzania, 63% of adults were receiving antiretroviral treatment equating to 792,000 adults increasing from 688,600 in 2015. In Kilimanjaro region 21,601 adults ≥15 years of age were receiving ART provided from 278 health facilities.^8^ It is estimated that 21–30%of people living with HIV (PLHIV) have been registered in care and treatment clinics (CTC) and of these 69% of eligible adults are receiving ARVs.^9^ However very little information regarding adverse drug events is reported to the National Aids Control Programme (NACP) to guide management of patients at local CTCs.^9^

Pharmacovigilance is a useful tool to ensure the safety of drugs and protect patients from their harmful effects. Strategies and policies for pharmacovigilance are more advanced in countries with more developed health systems. In more developed health systems pharmacovigilance is maintained by the reporting of all suspected ADRs and unexpected events to drug safety through website, emails, fax, and known electronic data base to the relevant authorities.^11^ In Africa, pharmacovigilance has been progressively growing and its importance in the health-care system is being increasingly realised.^12^ Previous studies suggest that HCPs were not reporting due to inadequate knowledge on how to fill the ADR forms and the unavailability of the reporting forms.^10^ In 1993, Tanzania officially became a member of the WHO international drug monitoring programme. However, to date ADR reporting rates in Tanzania remain suboptimal. The required WHO standard is 200 ADR reports per million inhabitants per year.^18–20^ Recent studies showed that the average reporting rate was 3 reports per million inhabitants per year (0–21), where as Tanzania had only reported 400 ADR which equates to just 1 report per million inhabitants per year. Such low rates suggest an urgent need for increasing awareness and reviewing the reporting systems to ensure ADR takes place. A study in Tanzania showed that lack of awareness among health-care providers affects the pharmacovigilance reporting system.^14^

Adequate reporting of ADR is required in order for Tanzania to achieve the third sustainable development goal addressing healthy wellbeing of all people. There are on-going efforts to improve pharmacovigilance systems in Tanzania. In October 2016, a new web based system-electronic system was launched to complement the existing reporting forms.^15^

All health-care providers are required to report adverse drug reaction as part of their professional obligation in their countries globally.^3^ According to TFDA, all health-care providers including specialists, doctors, dentists, pharmacists and nurses should report ADRs. All affected consumers are encouraged to report ADRs directly to their health-care professionals and zonal drug information centres or can self-report using the green reporting forms. This is accomplished through special forms for reporting ADRs (yellow forms) that should be filled by health-care professionals and sent to the National Centre for ADRs monitoring under the Tanzania Food and Drugs Authority (TFDA). In Tanzania these forms are available in CTC centres. The TFDA is responsible for monitoring pharmacovigilance activities. It is important to report an ADR even if all the facts are not available or if the reporter is uncertain that the drug is definitely responsible for causing the reaction.^16^

In 2006, a sensitisation tool delineating the importance of the yellow card and how to fill was developed at Kilimanjaro Christian Medical Centre and the number of reported ADR was increased to a total of 105 cases between May 2006 and August 2007 at KCMC and 7 months later declined.^17^ Studies are needed to understand why ADR has once again reduced. In this study we propose to determine awareness and asses the roles of patients and health-care professionals in their contribution to pharmacovigilance activities so as to reduce a considerable degree of under estimating safety related issues pertaining to ART.

## METHODS

### Study Design and Study Setting

A cross sectional questionnaire-based study was conducted from June to July 2016 at the main CTC and dermatology clinics at KCMC. KCMC is a consultant and teaching hospital in Northern part of Tanzania, serving about 15 million inhabitants, mainly from the north eastern zone covering Tanga, Kilimanjaro, Arusha, Manyara and Singida. It is among the first 4 sites in Tanzania to offer free ARVs since September 2004 and approximately 70 to 90 patients are seen per week for their monthly clinical visits.

### Study Population, Sample Size, and Sampling Procedure

The study population included both ART patients and health-care providers. HIV/AIDS patients registered at KCMC and have been taking ARTs for at least 1 year at the same clinic.

All health-care providers including doctors, nurses and pharmaceutical technologists involved in HIV care and treatment and had a minimum of 1 year experience were eligible to take part in the study. All health-care providers who consented were included in the current study.

### Study variables

The main outcome measures were knowledge about ADRs and practice of ADRs reporting (report or not report). The independent variables for the patients included; demographic characteristics (age, sex, marital status, address), level of education and duration on ART

The independent variables for health-care providers included; demographic characteristics (age, sex, marital status, address), level of education/qualification of HCP, years of work experience, training on pharmacovigilance, patient load and availability of reporting forms.

### Data Collection Tools and Methods

A standard questionnaires adapted from earlier studies was administered. The questionnaire collected data on the socio demographics of the patients, knowledge of patients and HCP, practices of health HCP about ADR reporting and factors affecting reporting of ADRs. Specifically the questionnaire included a series of questions adapted and adopted from earlier studies to assess the knowledge, practices and factors affecting ADR reporting among patients and medical practitioners.^18–20^ For ART patients, the questionnaire administered into Swahili by a trained researcher, health-care providers self-completed an English version of the questionnaire.

### Data Processing and Analysis

Each day all questionnaires were double checked by MK to minimise errors and quality control. Then data were entered and cleaned in SPSS (IBM SPSS Statistics for Windows version 20.0 (IBM Corp, Armonk, NY, USA). Data was then transferred to Stata version 13 (Stata Corp LLC Durham, United Kingdom) for analysis. Mean and standard deviation was used to summarise the numerical data with normal distribution while undistributed numerical data were summarised using median and range. Frequency and percentages were used to summarise categorical variables.

Odds ratios (ORs) and 95% confidence intervals were calculated to determine the strength of association between outcomes and independent predictors. *P*<.05 was considered statistically significant.

### Ethical Considerations

The study protocol was approved by the Kilimanjaro Christian Medical University College Research Ethics and Review Committee (CRERC), in Moshi, Tanzania (certificate number: 2396). Permission to carry out the study was obtained from KCMC hospital management. Informed consent was obtained from all study participants and the health-care providers.

## RESULTS

### Study Participants

Of 63 health-care providers that were enrolled in the study, 20 (31.7%) were male and 43 (68.3%) were female. Five (7.9%) specialists, 17 (27%) residents, 10 (15.9%) assistant medical officers, 11 (17.5%) pharmaceutical technologists, and 20 (31.7%) nursing officers (Table 1).

**TABLE 1.** Health-Care Worker Characteristics (N=63)

Variable	n (%)
**Job title**	
Specialist	5 (7.9)
Resident	17 (27)
Assistant medical officer	10 (15.9)
Pharmaceutical technologists	11 (17.5)
Nursing officer	20 (31.7)
**Sex**	
Male	20 (31.7)
Female	43 (68.3)
**Work experience, years**	
<5	20 (31.7)
>5	43 (68.3)
**Training on pharmacovigilance**	
Yes	32 (50.8)
No	31 (49.2)

Out of 193 patients interviewed, majority were females 132 (68.4%) and males 61 (31.6%) with the mean age 48.4±10.6 years. Potential interviewees were approached when they came to the hospital for their monthly refills.

### Knowledge of Patients About ART and ADRs

About 153 (79.3%) of the patients did not know the names of the drugs they were taking. However most patients (n=184, 95.3%) only recognised drugs by either shape or colour. Ninety-five (49.2%) thought that all medicines caused unpleasant effects, 44 (22.8%) did not think so while 54 (28%) did not know if medicines can cause unpleasant effects. The majority (n=111, 57.5%) of patients on ART reported to not have ever experienced ADR, 64 (33.2%) had experienced, and only 18 (9.3%) did not know if they had experienced or not. More than two-thirds (n=134, 69.4%) reported to have been counselled on ART ADR by providers. Of these, 106 (78.5%) were counselled before starting medication and 29 (21.5%) after the appearance of ADR. Fifty-eight (30.1%) had never been counselled on ART ADR. However, most patients (n=142, 73.6%) reported knowing what to do when they experience ADR, which included reporting to health-care provider.

### Knowledge of HIV/AIDS Health-Care Providers on ADR Reporting

**FIGURE 1.**
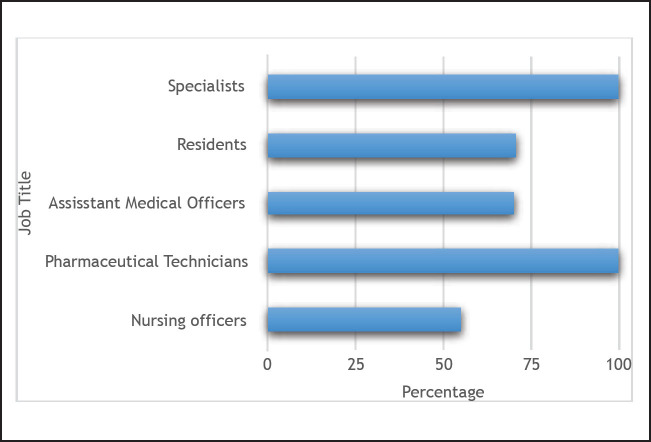
Awareness of Health-Care Workers About the Existence of a National Adverse Drug Reaction Reporting System

Forty-six (73%) of the health-care providers knew of the existence of national ADR reporting system. These were primarily the specialists and pharmaceutical technicians (n=5, 100%) with the lowest awareness amongst nursing officers (n=20, 55.0 %), see Figure 1.

**FIGURE 2.**
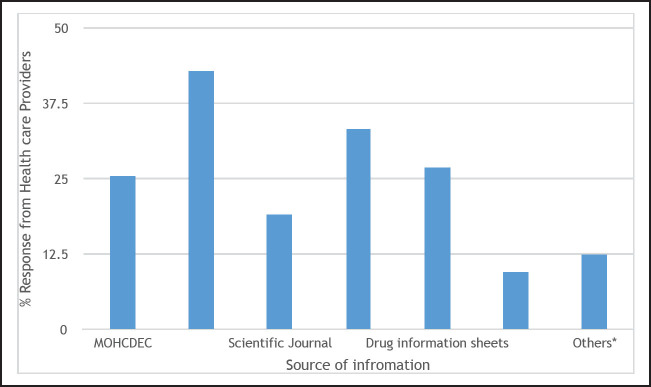
Sources of Information About Adverse Drug Reactions

Abbreviations: MOHCDEC, Ministry of Health, Community Development, Gender, Elderly, and Children; TFDA, Tanzania Food and Drug Authority *From patients, hospital pharmacists, Medscape, pharmacovigilance training, and special forms from pharmacy

**FIGURE 3.**
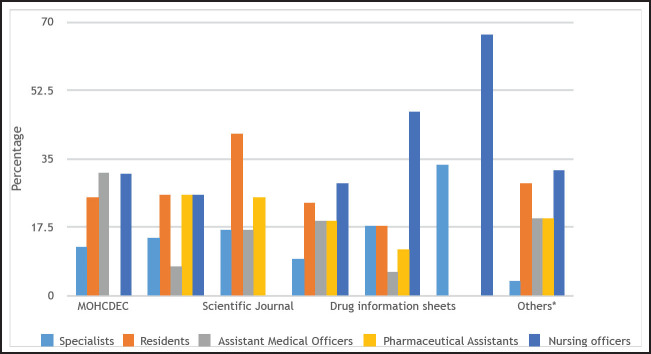
Percentage Compound Bar Graphs Illustrating the Proportion of Responses Regarding Sources of Information About Adverse Drug Reactions by Job Title (N=63)

Abbreviations: MOHCDEC, Ministry of Health, Community Development, Gender, Elderly, and Children; TFDA, Tanzania Food and Drug Authority *From patients, hospital pharmacists, Medscape, pharmacovigilance training, and special forms from pharmacy

About half of all health-care providers (n=32, 50.8%) reported to have had training on pharmacovigilance which included topics on ADR monitoring. When asked if they knew the ADR reporting forms (yellow forms), the majority (n=50, 79.4%) reported to know them. Of those who knew, more than three-quarters (82.0%) said the completed forms are sent to TFDA, 7 (14.0%) said the forms should go to the Ministry of Health, Community Development, Gender, Elderly and Children (MoHCDEC) and only 2 (4%) did not know where the forms are supposed to be sent. All respondents had a positive attitude on ADR reporting; all heath providers interviewed agreed that ADR reporting is necessary, 57 (90.5%) knew that reporting is their professional obligation and 54 (85.7%) knew that all health providers are responsible for ADR reporting. Twenty-seven (43%) reported TFDA as their sources of information on ADR, 21 (33.3%) reported they received information via texts on drugs and therapies, and 16 (25.4%) said they received information directly from the MoHCDEC, see Figures 2 and 3. Others reported tp receive information from patients, thepharmacy department, the internet (Medscape) and from pharmacovigilance training.

### Pharmacovigilance Practices of HIV/AIDS Care Providers

The majority of providers (n=39, 61.9%) reported to always counsel/provide information to their patients on possible ART ADR, 20 (31.7%) rarely and 4 (6.3 %) had never counselled their patients on possible ART adverse events. Only 4 (6.3%) of health-care providers reported to always fill ADR report forms, 27 (42.9%) rarely fill the forms and the majority (n=32, 50.8%) reported to have never filled an ADR report form, see Table 2. Of those that filled an ADR report form only 3 (9.7%) received feedback from TFDA and 28 (90.3%) reported to have never received any feedback from TFDA.

**TABLE 2.** Practices of Health-Care Workers Towards Pharmacovigilance Practice (N=63)

Variable	n (%)
**How often do you provide information on ADRs to patients?**	
Always	39 (61.9%)
Rarely	20 (31.7%)
Never	4 (6.3%)
**How often do you report ADRs to the adverse report forms?**	
Always	4 (6.3%)
Rarely	27 (42.9%)
Never	32 (50.8%)

Abbreviation: ADRs, adverse drug reactions

### Factors Discouraging Health-Care Providers From Filling ADR Forms

Respondents were asked on factors that discouraged them from filling the ADR report forms as health-care providers. Less than half of the respondents (n=23, 36.5%) lacked motivation, 19 (30.2%) were unsure on how to report, and 18 (28.6%) had no to time report. Other respondents reported unavailability of reporting forms (n=17, 27%), seriousness of the reaction (n=15,23.8%) and difficulty in deciding whether an ADR has occurred or not (n=13, 20.6%). Twelve participants (19%) stated that reporting creates an additional work load, 8 (12.7%) said that they didn't know that ADR should be reported 9 (14.3%) felt that a single ADR case does not affect the database. The following quotes illustrate other factors identified by some respondents individually concerning reporting of ART adverse effects:

*It hasn't been emphasised/sensitised and even the forms are not readily available and hence simply forgetting* (Health-care worker [HCW] 56, Female)

*Lack of feedback after filling and sending the forms* (HCW 55, Female)

*Nobody has ever mentioned to me that ADR should be reported* (HCW 47, Female)

*No known ADR monitoring centre in the hospital and poor channel of reporting* (HCW 15, Male)

### ADR Monitoring and Reporting Among Health-Care Providers

Health-care providers were asked for suggestions to improve ADR reporting. The majority (n=53, 84.1%) suggested improving awareness through workshops and clinical conferences, 45 (71.4%) requested the provision of reporting guidelines and reporting forms by TFDA, 26 (41.3%) suggested that reporting should be done by cell phones fax, emails and electronic data base, 39 (61.9%) felt that pharmacovigilance should be taught in detail to healt-care professionals, 48 (76.2%) recommended establishing ADR monitoring centres in every hospital, region, and zone. Some health-care providers suggested educating patients on ADRs. Other health-care providers recommended the need to identify a focal person within every department to receive the forms. However, others reported that, even though the hospital pharmacist is supposed to be responsible for receiving all the forms, in practice this does not happen and reporting is still low. The quotes below illustrate other suggested ways to improve pharmacovigilance practice among health-care providers:

*Every patient/drug user should be educated on the importance of reporting an adverse reaction* (HCW 36, Female)

*Identification of focal person at every department and sharing experience annually* (HCW 20, Female)

*Incorporate ADR monitoring in MD, Pharmacy and nursing training programme* (HCW 47, Female)

*Motivation to anyone who reports ADR and regular training of staff dealing drugs* (HCW 38, Female)

*To have specific focal person receiving all forms* (HCW 51, male)

*Use of media and add ways to report herbal ADR* (HCW 6, male)

## DISCUSSION

In the era of widespread ART for all persons living with HIV, it is clear that adverse drug reactions are going to exist. It is now, more pertinent, than ever that reporting systems are in place to ensure adverse reactions are captured and monitored on a national level. This study provides a case study to illustrate the knowledge and practice of health-care providers towards adverse drug reaction reporting in a tertiary health facility in Northern Tanzania. This study revealed that even though majority of the health-care providers had a positive attitude towards ADR reporting, awareness was low among nursing officers.^21^ Our data suggest that health-care providers who had training in pharmacovigilance were better at reporting ADR when compared to those not trained. However, the low awareness amongst nurses poses and important challenge. In a resource limited and high prevelance settings, like Tanzania, nurses provide the majority of patient care. Whilst clinically trained providers may be the ones to initiate ART, in the era of Universal test and treat, more and more clinically trained lower cadre personnel are also responsible for ART delivery. It is of paramount importance that these personnel are able to firstly detect ADR and then secondly to know the processes to follow to ensure proper reporting and action is taken.

Training health-care providers on pharmacovigilance is, therefore, an important factor in influencing good pharmacovigilance practices among health providers.^1^

In order to address some of the factors of under-reporting found in this study, ADR sensitisation workshops and presentations at the health-care facilities should be done every 6 months. Also posters at conspicuous locations in health-care facilities to serve as a constant reminder and establishment of ADR monitoring centre with a specified focal person in every hospital.

Creating awareness and promoting self-reporting among our patients will improve ADR reporting, and potentially help to reduce their health-care costs. The patients self-reporting has shown a complimentary role in increasing the number of ADR reporting and the benefits of this idea have been confirmed in different studies.^25,26^

The main limitation of our study was the relatively small number of health-care providers and factors that were associated with self-reporting, such as accuracy of recall and personal bias that might in some ways affected the results of this study.

## CONCLUSION

The majority of health-care providers were aware of ADR reporting and national pharmacovigilance systems however the results of this study suggests there is underreporting of ART adverse events. Our study suggests that most HCP did not have good practice towards pharmacovigilance. It is therefore important that factors discouraging reporting among HCW should be given special attention and resolved so as to improve pharmacovigilance practice and ADR reporting in our hospitals. There is a need for regular sensitisation workshops and training for ADR reporting among health-care providers.
